# Chromoblastomycosis caused by *Alternaria infectoria*, concurrent with myiasis, in a recipient of a kidney transplant: a compelling case report

**DOI:** 10.3389/fmed.2024.1396224

**Published:** 2024-07-16

**Authors:** Hamidreza Mahmoudi, Zahra Ramezanalipour, Mahmoud Khansari, Eelco F. J. Meijer, Shahram Mahmoudi, Bram Spruijtenburg, Abbas Rahimi Foroushani, Mohsen Gramishoar, Hasti Kamali Sarvestani

**Affiliations:** ^1^Autoimmune Bullous Diseases Research Center, Tehran University of Medical Sciences, Tehran, Iran; ^2^Department of Medical Parasitology and Mycology, School of Public Health, Tehran University of Medical Sciences, Tehran, Iran; ^3^Department of Surgery, Sina Hospital, Tehran University of Medical Sciences, Tehran, Iran; ^4^Radboudumc-CWZ Center of Expertise for Mycology, Nijmegen, Netherlands; ^5^Canisius-Wilhelmina Hospital (CWZ)/Dicoon, Nijmegen, Netherlands; ^6^Department of Parasitology and Mycology, School of Medicine, Iran University of Medical Sciences, Tehran, Iran; ^7^Department of Epidemiology and Biostatistics, School of Health, Tehran University of Medical Sciences, Tehran, Iran

**Keywords:** chromoblastomycosis, mycoses, *Alternaria*, organ transplantation, neglected diseases

## Abstract

Neglected tropical diseases (NTDs) pose a significant threat to the health of millions of people worldwide, particularly in impoverished populations in tropical and subtropical regions. The World Health Organization (WHO) considers certain fungal infections, such as chromoblastomycosis, as NTDs. Chromoblastomycosis is a chronic fungal infection affecting the skin and subcutaneous tissue, primarily found in tropical and subtropical regions of Latin America, Africa, and Asia. This case report presents a 46-year-old female patient with chromoblastomycosis who had a history of renal transplantation and was receiving immunosuppressive therapy. The patient exhibited dark, verrucous, and ulcerative lesions on the legs, and the diagnosis was confirmed through the microscopic examination of skin scrapings by observing medlar bodies. Two sequential fungal tissue cultures and ITS sequencing verified the presence of *Alternaria infectoria*, not formerly described in chromoblastomycosis. Moreover, observation of fly larvae in the lesions verified the diagnosis of myiasis. Treatment with voriconazole and terbinafine resulted in complete resolution of the lesions after 5 months. This case emphasizes the importance of considering chromoblastomycosis in individuals with occupational exposure in tropical areas, as well as the challenges associated with its diagnosis, coinfections, and treatment.

## Introduction

Neglected tropical diseases (NTDs) pose a significant threat to the health of millions worldwide, particularly affecting impoverished communities in tropical and subtropical regions. According to the World Health Organization (WHO) report, NTDs encompass several endemic diseases, including parasitic, bacterial, viral, and fungal infections. One example of NTD is Chromoblastomycosis (1–3).

Chromoblastomycosis, also known as chromomycosis, is a chronic fungal infection that affects the skin and subcutaneous tissues. This infection could be caused by a variety of dematiaceous fungi, typically species of *Fonsecaea* and *Cladophialophora*. Due to its rarity, the exact prevalence of chromoblastomycosis remains unknown. However, it is more commonly reported in tropical and subtropical regions such as Latin America, Africa, and Asia. South America, particularly Venezuela, Brazil, and Colombia, bears the brunt of the impact, while in Africa, Madagascar, South Africa, the Republic of the Congo, and the Democratic Republic of the Congo are among the most affected nations. Central America, including Mexico, the Dominican Republic, and Cuba, also reports a high number of cases. Asia also sees significant hotspots, notably in China, Japan, and India (4). The fungi responsible for chromoblastomycosis are found in soil, wood, and decaying vegetation (5, 6). Therefore, individuals engaged in agriculture, forestry, and other outdoor activities are at a higher risk of contracting the disease (4, 7).

Chromoblastomycosis is a slow-progressing fungal skin disease that begins as a small nodule and develops into a warty, plaque-like lesion. It may take months or even years to spread and appear brown or black. The condition can become painful and lead to complications such as cellulitis and secondary bacterial infections (8).

Diagnosis involves clinical examination, fungal culture, histopathology, and molecular techniques. Microscopy is essential, with sclerotic bodies being a characteristic feature in histological sections stained with Hematoxylin and eosin or Gomori methenamine silver (1, 9, 10). Other fungal structures, such as hyphae, conidia, or chains of conidia, may also be observed, depending on the species of the fungus (11).

The successful treatment of chromoblastomycosis often consists of long-term antifungal therapy with medications such as itraconazole, terbinafine, or voriconazole. In some cases, surgical excision of the lesion may also be necessary (11).

Chromoblastomycosis is a rare condition in Iran with only two reported cases (12, 13). Our report highlights another case of this infection from Iran.

## Case presentation

**Figure 1:**
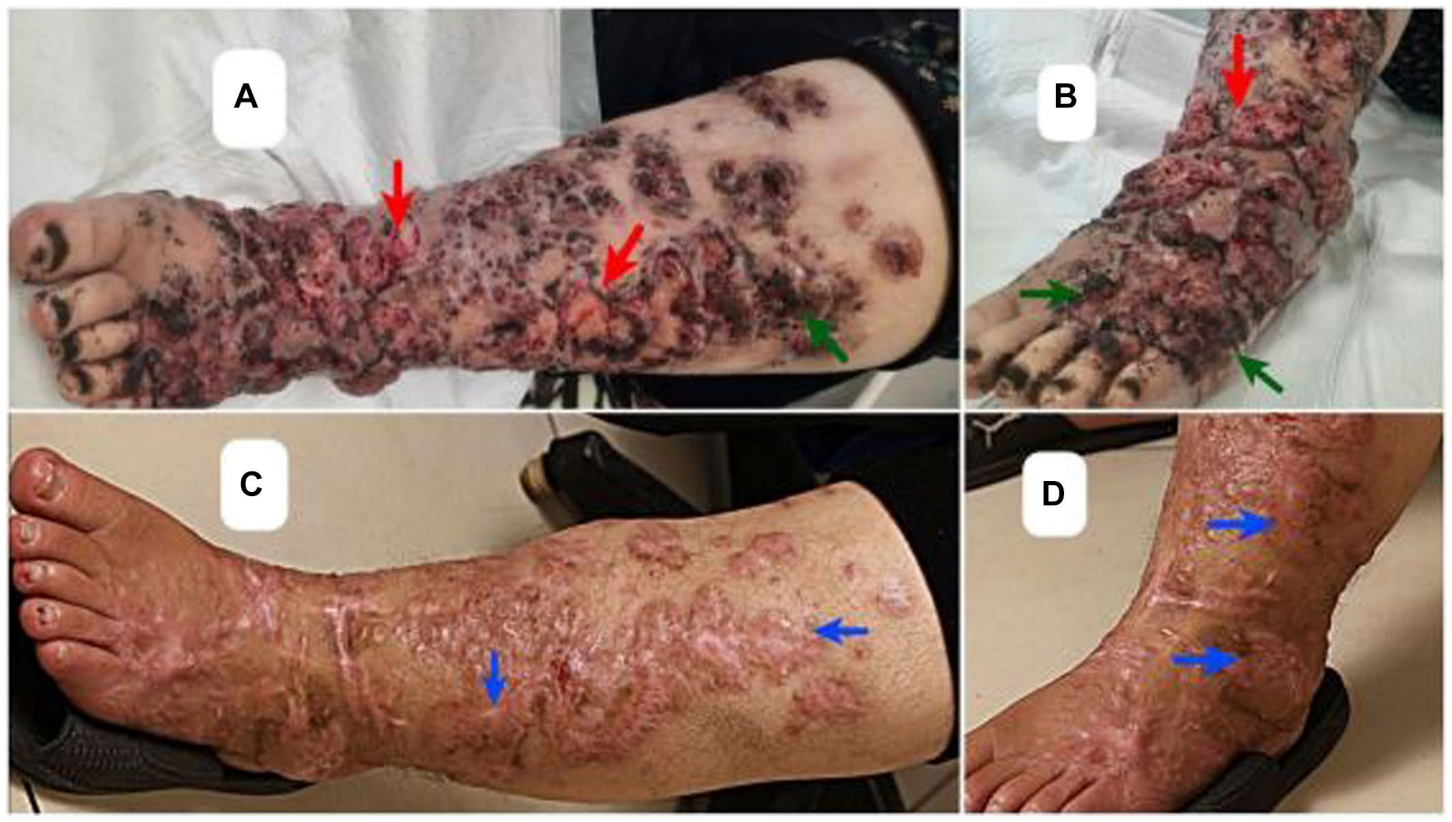
Clinical presentation of pigmented hyperkeratotic scaly plaques (green arrows) and verrucous ulcerated lessons on legs (red arrows) **(A,B)**, and the scars of healed Chromoblastomycosis plaques (blue arrows) post therapy **(C,D)**.

A 46-year-old woman with a history of renal transplant 8 years ago, presented with dark, verrucous, and ulcerative lesions on both legs (Figures 1A,B). The patient received a renal transplant due to end-stage renal disease (ESRD) stemming from chronic kidney disease (CKD) and she also had diabetes mellitus, which is a known risk factor for CKD and ESRD. Following the transplant, she was taking immunosuppressive medications, including mycophenolate mofetil at 1,000 mg/day, prednisolone at 5 mg/day, and tacrolimus at 2 mg/day. The emergence of lesions coincided with ulceration 1 year post-transplant. Initially Seeking treatment in Ahvaz, Khuzestan, the patient, with an occupation in agricultural work in neighboring villages, underwent a biopsy and received a four-month itraconazole (100 mg bd) treatment as well as multiple cryotherapy sessions, all without improvement., Consequently, recognizing the persistence and worsening of the condition, she pursued advanced medical care in Tehran, where hospitalization and further treatment were provided.

The patient’s medical records revealed that she had undergone a kidney transplant and was using immunosuppressive drugs, including 5 mg/day of prednisolone, oral tacrolimus, and mycophenolate mofetil. There were no similar lesions found on other parts of the body, and despite a slight fever (37.9°C), her overall condition was satisfactory.

**Figure 2:**
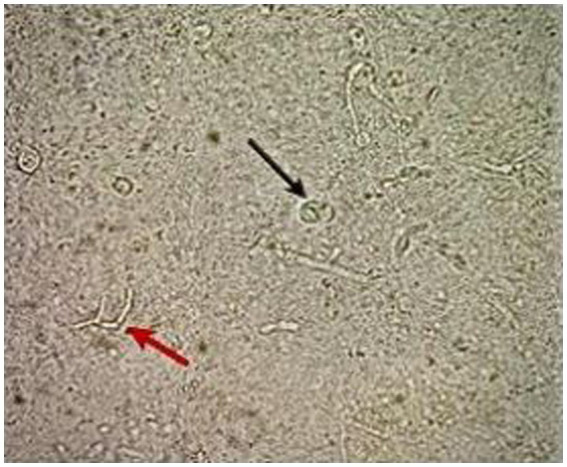
Direct microscopy using KOH 10% shows the thick-walled ovoid sclerotic cells (black arrow) along with septate hyphae (red arrow).

During the follow-up physical examination, inflammatory, nodular, ulcerative, disseminated, and hemorrhagic lesions were found on the patient’s legs. These lesions were distributed across the patient’s legs. Upon further assessment, microscopic examination of skin scrapings using potassium hydroxide (KOH) revealed the presence of septate hyphae and ovoid bodies with thick walls, known sclerotic or medlar bodies (Figure 2). X-rays targeting potential bone involvement were not conducted because there were no clinical indications at the time, according to the patient’s records. The attending physicians observed no signs of bone involvement, so further radiological follow-up was deemed unnecessary.

**Figure 3:**
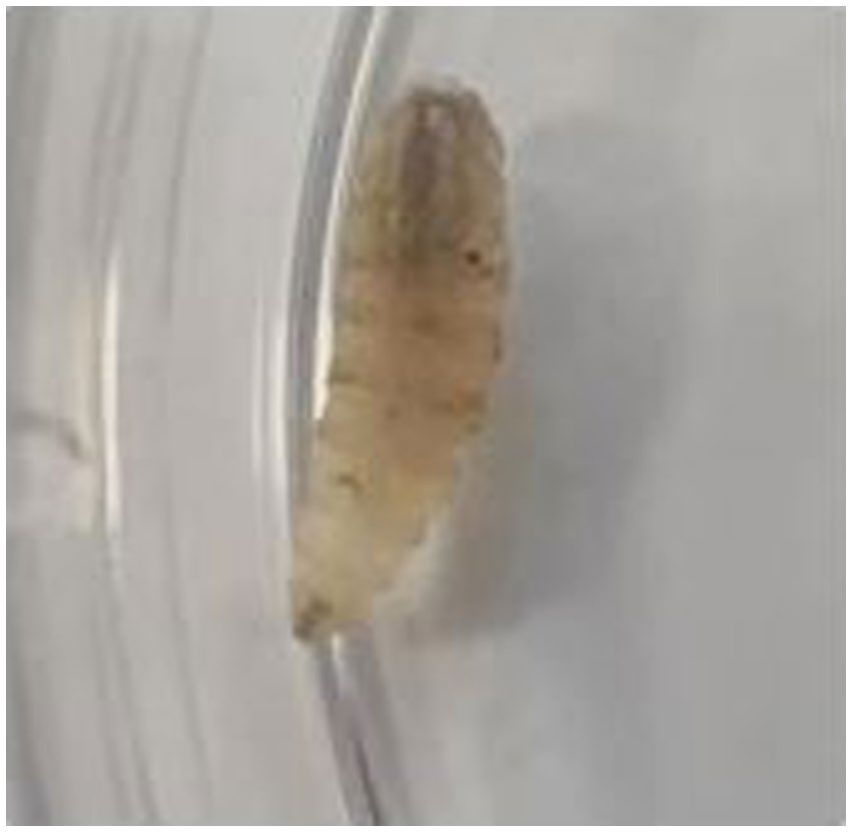
Presence of fly larvae within the lesions, confirming the diagnosis of myiasis.

**Figure 4:**
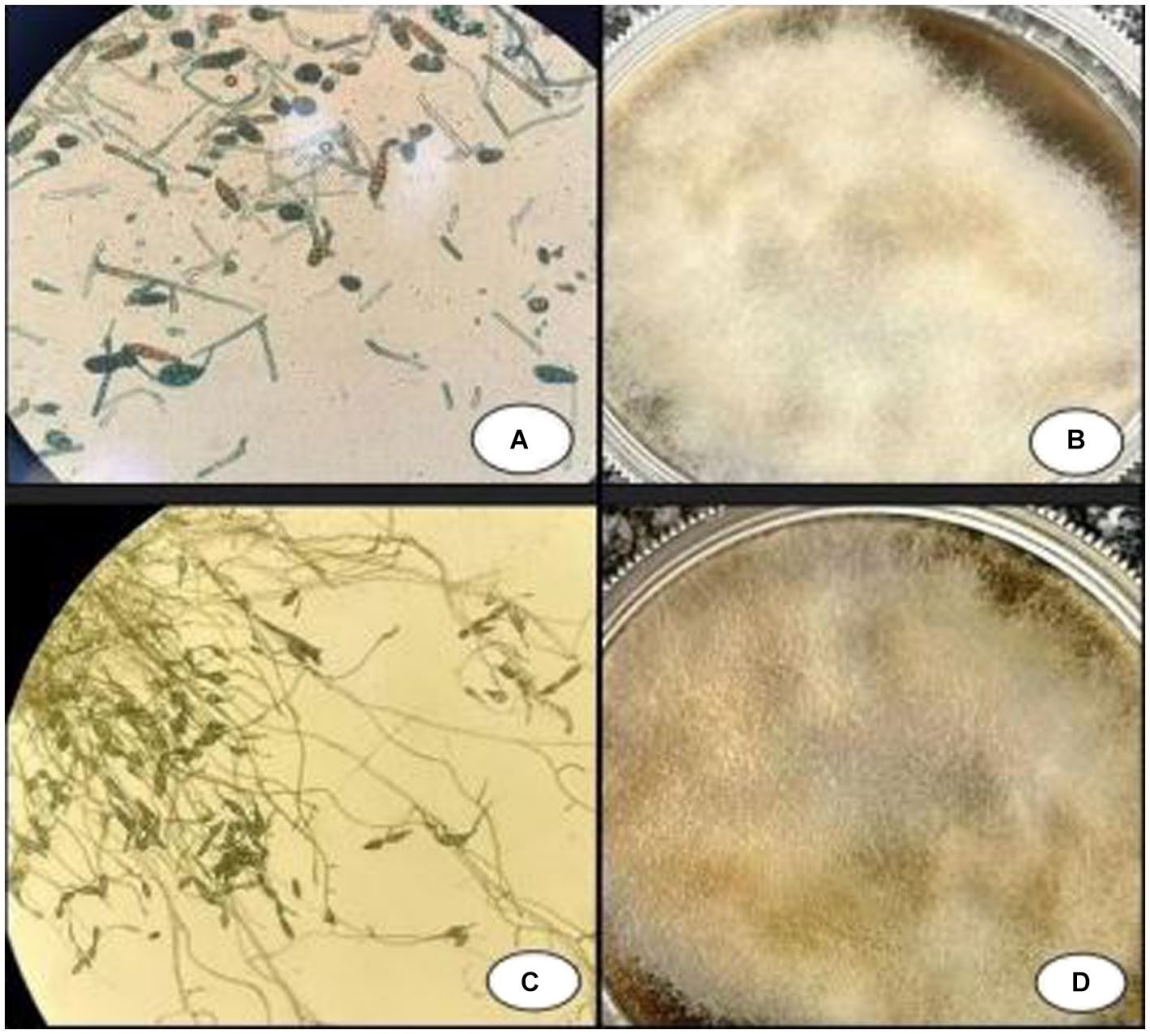
Pigmented septate hyphae with chains of ovate conidia with transverse and vertical septa: **(A)** sample from week 1 and **(C)** sample from week 2. Colonies of *Alternaria* grown on SDA with chloramphenicol media: **(B)** sample from week 1 and **(D)** sample from week 2.

**Figure 5:**
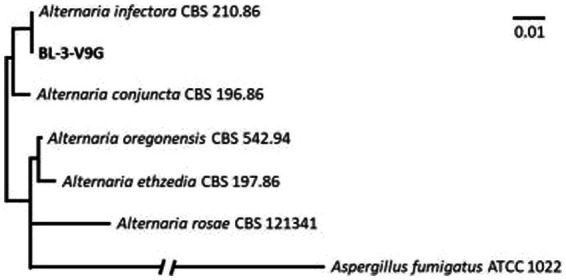
The phylogenetic tree generated with Clustal Omga using the Multiple Alignment Algorithm.

We successfully isolated a positive culture from the lesions on the patient’s foot, followed by another positive culture from the lesions 1 week later. Notably, the presence of fly larvae in the lesions also led to a diagnosis of myiasis (Figure 3). Tissue specimens were cultured on Sabouraud dextrose agar (SDA, Merck, Germany) supplemented with chloramphenicol (QUELAB, UK) and were incubated at 30°C for 20 days. On the ninth day of incubation, we observed some grayish colonies on the culture medium. Microscopic examination revealed pigmented septate hyphae along with chains of ovate conidia possessing both transverse and vertical septa. This confirmed the identity of the isolate as *Alternaria* species, demonstrating that both positive cultures were consistent with *Alternaria* species (Figure 4). Subsequent species identification was accomplished via internal transcribed spacer (ITS) sequence analysis. Genomic DNA was isolated from fresh colonies using a previously described method (14). The ITS rDNA regions were amplified using 0.25 μM of the fungal universal primers V9G and LS266, 12.5 μL of 2× premix (Ampliqon, Denmark), 1 μL of DNA template, and enough water to produce a final volume of a 25 μL reaction mixture (15). The generated ITS sequence was deposited under Genbank accession number OR685707. ITS sequences of *Alternaria infectoria* CBS 210.86 (NR_131263.1), *Alternaria conjuncta* CBS 196.86 (NR_135929.1), *Alternaria rosae* CBS 121341 (NR_136017.1), *Alternaria oregonensis* CBS 542.94 (NR_135935.1), and *Alternaria ethzedia* CBS 197.86 (NR_135928.1) were used in phylogenetic analysis and *Aspergillus fumigatus* ATCC 1022 (NR_121481.1) was taken as an out-group. A phylogenetic tree was generated with Clustal Omga using the Multiple Alignment Algorithm (16) (Figure 5).

After conducting dermoscopic and microscopic examinations, as well as the result of ITS sequencing, the lesions were determined to be different from deep mycosis, atopic mycobacterium, TB, leishmaniasis, pyoderma gangrenosum, and vasculitis.

Microdilution testing was performed following the methodology outlined by the European Committee on Antimicrobial Susceptibility Testing (EUCAST) (17). The antifungal agents used in this study included amphotericin B (Sigma-Aldrich, United States), voriconazole (Sigma-Aldrich, United States), and terbinafine (Combi-Blocks, United States). The endpoint for evaluation was the antifungal concentration that resulted in complete inhibition of visible growth at 48 h (MIC). According to the clinical breakpoints and epidemiological cut-off values set by EUCAST (18), the isolate demonstrated susceptibility to all three antifungals, exhibiting MIC values of 0.5 mg/L for amphotericin B, 0.5 mg/L for voriconazole, and 0.03 mg/L for terbinafine. Initially, intravenous conventional amphotericin B 1 mg/kg/day for 15 days was selected for the therapy. However, due to the patient’s history of seizures following an eight-day course of amphotericin B treatment, a revised approach to the treatment was undertaken. The patient was administered voriconazole at a daily dose of 200 mg, without therapeutic drug monitoring. The initial prescription of terbinafine was 250 mg/day, which was subsequently reduced to 125 mg/day after a month and a half. After 5 months of this regimen, complete resolution of the lesions was achieved confirmed via negative cultures and clinical improvement of the healed lesions (Figures 1C,D).

## Discussion

Chromoblastomycosis is a chronic granulomatous disease that arises as a result of subcutaneous inoculation of fungal elements (1). This infection manifests in various forms, necessitating accurate identification of fungal elements for diagnosis. Initially, it begins as a small nodule or papule on the skin, gradually evolving into a wart-like or plaque-like appearance. Over time, the lesion enlarges, thickens, and develops scales, often taking months or even years to spread. The affected area commonly exhibits a brown or black coloration (8, 19). In this particular case, the presence of inflammatory, nodular, ulcerative, disseminated, and hemorrhagic lesions was observed. Typically, the condition predominantly affects young male workers (4) and farmers in developing countries, primarily due to their exposure to contaminated soil or plants (5, 7). The majority of chromoblastomycosis lesions occur on the lower extremities (7). This was the case with the first report of chromoblastomycosis from Iran, where the patient, a 26-year-old female worker, experienced leg involvement after being bitten by a leech while working in a paddy field. Due to the absence of any other symptoms causing discomfort aside from the formation of a painless and non-itchy black wart at the site of the wound, she did not seek medical attention until 4 years later, when a tumor-like lesion developed in the thigh of the same leg (12). In the second case from Iran, the patient had chest and palate involvement with no reported history of agricultural work. In this uncommon case of chromoblastomycosis, involved a healthy 27-year-old man who first noticed his condition approximately 11 years before its cause was identified. Initially, it appeared as a small pink lesion on the hard palate, accompanied by a scaly papule on the anterior chest. Despite multiple hospital admissions and various treatments, none were effective in curing the disease. Furthermore, all other medical tests yielded normal results, ruling out alternative causes besides fungi (13).

In our case, the patient was female with a typical agricultural background, which could have led to an inadvertent inoculation. Considering the patient’s history of agricultural work in rural areas, where individuals often work under challenging conditions of traditional agriculture to make a living, suggests that she may belong to a lower socioeconomic stratum.

In an interesting observation, fly larvae were found within the lesions, resulting in a diagnosis of myiasis. Myiasis, caused by infestation with fly larvae, could potentially serve as an entry point for fungal infection, particularly in immunocompromised individuals (20). The presence of open wounds or lesions associated with myiasis may potentially create an environment conducive to secondary fungal colonization, increasing the likelihood of developing conditions like chromoblastomycosis.

Diagnosing myiasis is usually easy when larvae are visible in the wound like this case. However, when larvae cannot be seen under the skin, ultrasound becomes a helpful diagnostic tool. It can accurately detect characteristic features of larvae, including their oval shape with a hypoechoic rim and hyperechoic center, spontaneous movements, and peripheral blood flow. Ultrasound aids in distinguishing myiasis from conditions like cysts and abscesses, facilitating the precise extraction of the parasite to ensure complete removal (21).

The principal causative agents responsible for this condition, as seen in previous cases reported from Iran, include *Fonsecaea pedrosoi, Cladosporium carrionii, Phialophora verrucosa,* and *Exophiala jeanselmei* (1, 12, 13, 22), all of these belong to the Herpotrichiellaceae family. The diagnosis of chromoblastomycosis is established through the identification of muriform cells in clinical samples (1, 9–11). In this case, confirmation was obtained solely by direct microscopy, revealing the presence of muriform bodies, which is pathognomonic for Chromoblastomycosis. Upon further examination of the culture results, it was discovered that *Alternaria* species were present. It is important to note that *Alternaria* species are common contaminants in laboratories and do not belong to the family Herpotrichiellaceae. As such, the true causative agent of the chromoblastomyces might not have been found and the obtained positive culture could have been mere contaminants. However, since these isolates were found sequentially from sterile biopsies two times, it’s confirmed that the infection was caused by *Alternaria*.

To our knowledge, this case represents the first instance in which *Alternaria infectoria* has been identified in a patient with chromoblastomycosis. More reports of *Alternaria* species from chromoblastomycosis are necessary to determine if this genus is capable of causing the disease. In the immunocompromised host, co-infection is possible. *Alternaria* is a widespread fungus found worldwide, commonly associated with various infections in humans, especially in immunocompromised individuals, where it can cause subcutaneous and invasive infections. Agricultural activities are a common pathway for the establishment of *Alternaria* species. These fungi, known for their cosmopolitan nature, thrive in environments rich in plant residues. Handling infected plants during agricultural tasks can facilitate the spread and establishment of *Alternaria* species. Consequently, the diagnosis of *Alternaria* infections relies on isolating the fungus in culture and simultaneously confirming the presence of fungal structures in tissues (23). Further, ITS sequencing in our study confirmed the presence of *Alternaria infectoria*. The notable aspect of this case is the patient’s history of renal transplant and the utilization of prednisolone, oral tacrolimus, and mycophenolate mofetil. These findings align with previous reports that indicate organ transplant and immunosuppressive therapy as contributing factors among chromoblastomycosis patients (24–26). With medical advancements, transplantation rates have increased, leading to a larger population of immunosuppressed individuals who are at higher risk of contracting opportunistic infections due to their weakened immune systems. There have been several cases of cutaneous involvement with *Alternaria infectoria* among kidney transplant patients worldwide with growing rates in recent years (27–34), but these cases have been reported as phaeohyphomycosis. Despite the similarity in underlying conditions, sites of infection, and clinical manifestations, phaeohyphomycosis caused by *Alternaria infectoria* typically presents as a cutaneous infection without sclerotic bodies. This is the first reported case in which this fungus is identified in a patient with chromoblastomycosis, which raises the question if this is a contamination or whether this could be a potential causative agent of chromoblastomycosis. Given that it does not belong to the family of Herpotrichiellaceae, the latter seems unlikely.

The treatment of chromoblastomycosis is challenging and requires a multidisciplinary approach involving dermatologists, infectious disease specialists, medical mycologists, and pathologists. The choice of antifungal agent depends on the severity of the disease, the specific fungal species involved, and the patient’s immune status. The commonly employed antifungal agents for this condition include itraconazole, terbinafine, and voriconazole. In some cases, surgical excision of the lesion becomes necessary (11). Previous cases in Iran have used amphotericin B, itraconazole along with surgical procedures (13), and, 5-fluorocytosine (12) for treatment, which were effective. In the present case, initial treatment involving itraconazole and cryosurgery was unsuccessful. Subsequently, voriconazole and terbinafine were selected for the patient’s treatment based on her favorable safety profiles and the results of antifungal susceptibility testing, leading to successful patient management.

In conclusion, this unique case highlights the potential risk of chromoblastomycosis for individuals living in tropical regions with occupational hazards and a history of organ transplantation. Treating this disease presents challenges, and successful management requires careful clinical differentiation and direct examination of clinical samples, especially when the fungal agent responsible is uncommon or there is a chance of contamination.

### Limitations of the study

The study’s reliance on a single case report makes it difficult to generalize the findings to a broader population. Chromoblastomycosis cases can vary significantly in terms of clinical presentation, underlying conditions, and treatment responses. Therefore, findings from a single case may not fully capture the spectrum of disease manifestations and treatment outcomes. It is crucial to conduct histopathological examination to differentiate Phaeohyphomycosis from Chromoblastomycosis; the decision not to employ the H&E staining method could be considered a limitation. From a histopathological perspective, the decision not to employ the H&E staining method could be regarded as a limitation. Longer-term follow-up data would provide a more comprehensive assessment of recurrence rates, and potential long-term complications associated with Chromoblastomycosis.

### Strengths of the study

This novel finding emphasizes the diversity of causative agents associated with chromoblastomycosis, broadening our understanding of the disease’s pathogenesis and epidemiology. Identifying *Alternaria* spp. as a potential causative agent emphasizes the importance of thorough diagnostic evaluation in cases where conventional etiological agents may not be implicated. The study employs a multidisciplinary diagnostic approach, including dermoscopic, microscopic, and molecular techniques, to accurately identify the causative agent of chromoblastomycosis. This comprehensive approach enhances the reliability of the diagnosis and contributes to the understanding of the disease pathology. The successful management of the patient’s condition with voriconazole and terbinafine highlights the importance of tailored treatment strategies in complex cases of chromoblastomycosis. The positive treatment outcome underscores the efficacy of the selected antifungal agents and provides valuable clinical insights for future cases.

## Data availability statement

The original contributions presented in the study are included in the article/supplementary material, further inquiries can be directed to the corresponding author/s.

## Ethics statement

Written informed consent was obtained from the individual(s) for the publication of any potentially identifiable images or data included in this article.

## Author contributions

HM: Investigation, Project administration, Supervision, Writing – review & editing. ZR: Writing – original draft. MK: Investigation, Writing – review & editing. EM: Writing – review & editing, Visualization. SM: Writing – review & editing. BS: Software, Visualization, Writing – review & editing. AR: Software, Visualization, Writing – review & editing. MG: Investigation, Writing – review & editing. HK: Investigation, Project administration, Supervision, Validation, Writing – review & editing.
